# The nicotinic acetylcholine receptor α7 subunit is an essential negative regulator of bone mass

**DOI:** 10.1038/srep45597

**Published:** 2017-03-28

**Authors:** Kazuaki Mito, Yuiko Sato, Tami Kobayashi, Kana Miyamoto, Eriko Nitta, Atsushi Iwama, Morio Matsumoto, Masaya Nakamura, Kazuki Sato, Takeshi Miyamoto

**Affiliations:** 1 Department of Orthopedic Surgery, Keio University School of Medicine, 35 Shinanomachi, Shinjuku-ku, Tokyo 160-8582, Japan; 2 Department of Musculoskeletal Reconstruction and Regeneration Surgery, Keio University School of Medicine, 35 Shinanomachi, Shinjuku-ku, Tokyo 160-8582, Japan; 3 Department of Advanced Therapy for Musculoskeletal Disorders, Keio University School of Medicine, 35 Shinano-machi, Shinjuku-ku, Tokyo 160-8582, Japan; 4 Department of Cellular and Molecular Medicine, Graduate School of Medicine, Chiba University, 1-8-1 Inohara, Chuo-ku, Chiba 260-8670, Japan

## Abstract

The nicotinic receptor α7nAchR reportedly regulates vagal nerve targets in brain and cardiac tissue. Here we show that *nAchR7*^−/−^ mice exhibit increased bone mass due to decreased osteoclast formation, accompanied by elevated osteoprotegerin/RANKL ratios in serum. Vagotomy in wild-type mice also significantly increased the serum osteoprotegerin/RANKL ratio, and elevated bone mass seen in *nAchR7*^−/−^ mice was reversed in α7nAchR/osteoprotegerin-doubly-deficient mice. α7nAchR loss significantly increased TNFα expression in Mac1-positive macrophages, and TNFα increased the osteoprotegerin/RANKL ratio in osteoblasts. Targeting TNFα in *nAchR7*^−/−^ mice normalized both serum osteoprotegerin/RANKL ratios and bone mass. Administration of nicotine, an α7nAchR ligand, to wild-type mice increased serum RANKL levels. Thus, vagal nerve stimulation of macrophages via α7nAchR regulates bone mass by modulating osteoclast formation.

Alpha 7 nicotinic acetylcholine receptor subunits form ligand-gated ion channels, which are permeable to Ca^2+^ and Na^+^ upon acetylcholine or nicotine binding. α7nAchR is abundantly expressed in hippocampus and implicated in the pathogenesis of Alzheimer’s disease; however, α7nAchR-deficient mice exhibit no obvious abnormalities in brain morphology or development^1^. As part of the parasympathetic nervous system, the vagus nerve regulates heart function; however, α7nAchR has not been shown to be required for parasympathetic control of cardiac activity^2^.

α7nAchR reportedly regulates inflammation via what is called the ‘cholinergic anti-inflammatory pathway’^3^,^4^,^5^. Previously, vagal nerve stimulation was shown to be anti-inflammatory^6^,^7^, as *α7nAchR*^−/−^ mice exhibit elevated levels of inflammatory cytokines^4^. Relative to wild-type (WT) controls, α7nAchR-deficient mice also exhibit severe joint destruction in a collagen-induced arthritis model^8^,^9^. There is less agreement in terms of bone phenotypes: some authors report that α7nAchR-deficient mice exhibit no obvious bone phenotypes^10^, while others demonstrate that these mice exhibit elevated bone mass^11^. However, a nAchR function in bone has not been fully characterized, despite the fact that smoking is a known risk factor for osteoporosis^12^,^13^,^14^ and nicotine is an α7nAchR ligand.

The cytokine tumor necrosis factor alpha (TNFα) promotes inflammatory diseases, such as rheumatoid arthritis (RA) and Crohn’s disease^15^. Inflammation is a known risk factor for osteoporosis^16^, and TNFα-overexpressing transgenic mice exhibit RA-like symptoms, arthritis and joint destruction due to osteoclast activation^17^, although a function for TNFα in increasing bone mass and inhibiting osteoclasts has not been reported.

Bone homeostasis is regulated by systemic and local signaling. In terms of the former, activation of the sympathetic nervous system reportedly inhibits bone formation in response to leptin signals^18^. The sympathetic nervous system also regulates osteoblastic function in hematopoietic stem cell egress^19^. Sensory nerve stimulation in bone reportedly promotes bone formation via the signaling factor Sema3a, and neuron-specific Sema3a-deficient and global Sema3a knockout mice exhibit altered osteoblast differentiation, elevated osteoclast function and reduced bone mass^20^,^21^. Meanwhile, local osteoclastogenesis increases following stimulation of progenitor cells by receptor activator of nuclear factor kappa B ligand (RANKL) produced by osteoblastic cells and osteocytes^22^,^23^. By contrast, osteoclastogenesis is inhibited by osteoprotegerin (OPG), a decoy receptor of RANKL, which is also produced by osteoblasts^24^. OPG is encoded by the *TNF receptor superfamily 11b (Tnfrsf11b)* gene, and *Tnfrsf11b*^−/−^ mice reportedly exhibit severe osteoporosis due to accelerated osteoclast formation^25^,^26^. In contrast, OPG-overexpressing transgenic mice show elevated bone mass due to inhibited osteoclastogenesis^27^, and RANKL (encoded by the *Tnfsf11* gene)-deficient mice exhibit severe osteopetrotic phenotypes and a complete absence of osteoclast formation^28^. Thus, the OPG/RANKL system plays a crucial role in maintaining bone homeostasis by regulating osteoclast differentiation. OPG expression in osteoblastic cells is down-regulated by treatment with active vitamin D3, 1,25(OH)_2_D_3_, while RANKL is up-regulated^24^. RANKL expression is stimulated by inflammatory cytokines such as TNFα and IL-6^29^.

Here, we show that α7nAchR is required to stimulate osteoclast formation and reduce bone mass by inhibiting circulating levels of OPG and elevating levels of RANKL. α7nAchR-deficient mice exhibited increased bone mass due to an elevated OPG/RANKL ratio in serum, and increased bone mass seen in α7nAchR-deficient mice was rescued in α7nAchR/OPG double-knockout mice. TNFα expression was aberrantly high in Mac1-positive macrophages among bone marrow cells in α7nAchR-deficient mice, and increases in bone mass and the OPG/RANKL ratio seen in α7nAchR-deficient mice were rescued in α7nAchR/TNFα double-knockout mice. Our results indicate overall that vagus nerve activity is required to maintain bone homeostasis by controlling systemic levels of OPG and RANKL via regulation of macrophage TNFα expression.

## Results

### Nicotinic acetylcholine receptor α7-deficient mice exhibit increased bone mass

**Figure 1:**
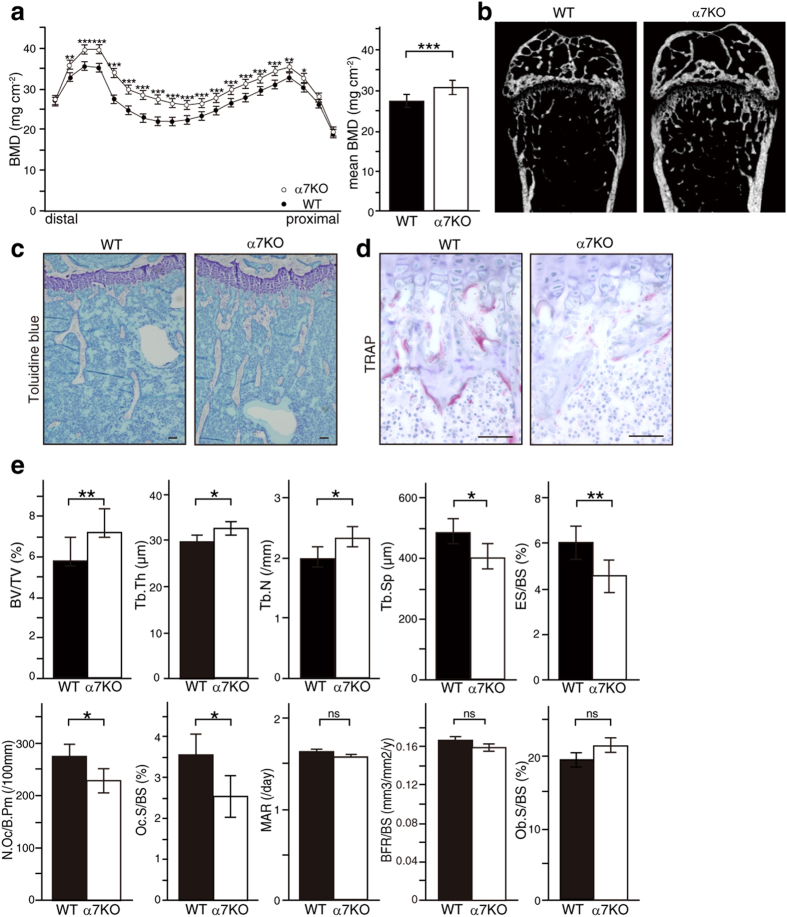
Osteoclastogenesis is inhibited in α7nAchR KO mice *in vivo*. (**a**) Bone mineral density (BMD) of femurs from of 8-week-old wild-type (WT) and α7nAchR KO (α7KO) mice equally divided longitudinally. Data represent means ± s.d of BMD (n = 9–10). (**b–d**) micro CT analysis and (**b**) toluidine blue (**c**) and TRAP (**d**) staining of WT and α7nAchR KO bones. Bar = 50 μm. (**e**) Bone morphogenetic analysis of 8-week-old WT and α7nAchR KO female mice. Data represent means ± s.d of bone volume per tissue volume (BV/TV), trabecular thickness (Tb.Th), trabecular number (Tb.N), trabecular separation (Tb.Sp), eroded surface per bone surface (ES/BS), osteoclast number per bone perimeter (N.Oc/B.Pm), osteoclast surface per bone surface (Oc.S/BS), mineral apposition rate (MAR) and bone formation rate per bone surface (BFR/BS) in indicated genotypes. Data represent means ± SD (*p < 0.05; **p < 0.01; ***p < 0.001; NS: not significant; *n* = 5).

α7nAchR is one of several subunits of nicotinic acetylcholine receptors^1^. Evaluation of bone phenotypes using DEXA and micro CT analysis revealed elevated bone mass in *α7nAchR*^−/−^ relative to WT mice (Fig. 1a and b). Toluidine blue and TRAP staining of tibial tissues from *α7nAchR*^−/−^ mice revealed elevated trabecular bone mass and inhibited osteoclast formation, respectively (Fig. 1c and d). Bone morphometric analysis also demonstrated significantly elevated bone volume per tissue volume (BV/TV), trabecular thickness (Tb. Th) and trabecular number (Tb. N) as well as significantly reduced trabecular separation (Tb. Sp) in *α7nAchR*^−/−^ compared with WT mice, confirming elevated bone mass seen in the mutants. Since osteoclastic parameters such as eroded surface per bone surface (ES/BS), number of osteoclasts per bone perimeter (N. Oc/B. Pm) and osteoclast surface per bone surface (Oc. S/BS) were significantly reduced in mutants, while osteoblastic parameters such as matrix apposition rate (MAR), bone formation rate (BFR) and osteoblast surface per bone surface (Ob. S/BS), were normal (Fig. 1e), we concluded that elevated bone mass in mutant mice resulted from inhibition of osteoclastogenesis.

**Figure 2:**
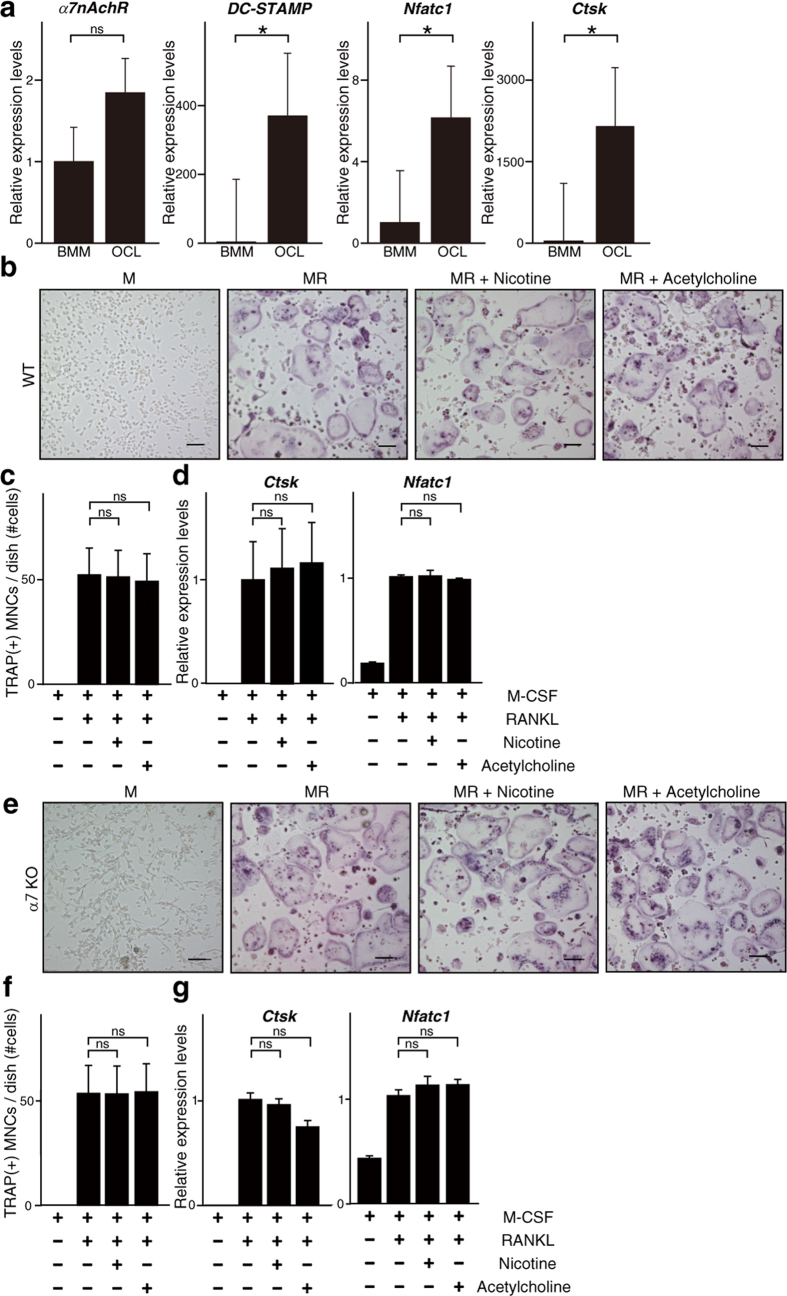
Osteoclastogenesis of progenitor cells is comparable in the presence of either α7nAchR-deficiency, nicotine or acetylcholine *in vitro*. (**a**) M-CSF-dependent osteoclast progenitor cells were isolated from wild-type mice and cultured in the presence of M-CSF (M, 50 ng/ml) + RANKL (R, 25 ng/ml). After five days, expression of *α7nAchR, DC-STAMP, Cathepsin K (Ctsk)* and *Nfatc1* was analyzed by realtime PCR. (**b**–**g**) M-CSF-dependent osteoclast progenitor cells were isolated from wild-type (WT, **b**–**d**) and α7nAchR KO (α7KO, **e**–**g**) mice and cultured in the presence of M-CSF (M, 50 ng/ml) + RANKL (R, 25 ng/ml), with or without nicotine (1 μM) or acetylcholine (1 μM) for 5 days. Cells were then stained with TRAP (**b** and **e**) (scale bar, 100 μm) and the number of multi-nuclear TRAP-positive cells (MNCs) was counted (**c** and **f**). Expression of *Cathepsin K (Ctsk)* and *Nfatc1*, both osteoclastic markers, was analyzed by realtime PCR (**d** and **g**). Data represent mean expression of each relative to *β-actin* ± SD (*p < 0.05; NS: not significant, *n* = 5). Representative data of at least two independent experiments are shown.

To determine whether α7nAchR regulates osteoclastogenesis directly, we isolated osteoclast progenitor cells from *α7nAchR*^−/−^ and WT mice and cultured them in the presence of M-CSF and RANKL, with or without the ligands acetylcholine or nicotine (Fig. 2). We found that α7nAchR was expressed in bone marrow macrophages (BMM) and osteoclasts (OCL) (Fig. 2a). However, osteoclastogenesis in WT cells was not stimulated by either ligand (Fig. 2b,c and d), and osteoclast formation was comparable in *α7nAchR*^−/−^ and WT cells (Fig. 2b–g). These observations suggest α7nAchR expressed in osteoclast progenitor cells does not regulate osteoclastogenesis directly.

### Vagal nerve activity regulates circulating OPG and RANKL levels via α7nAchR

**Figure 3:**
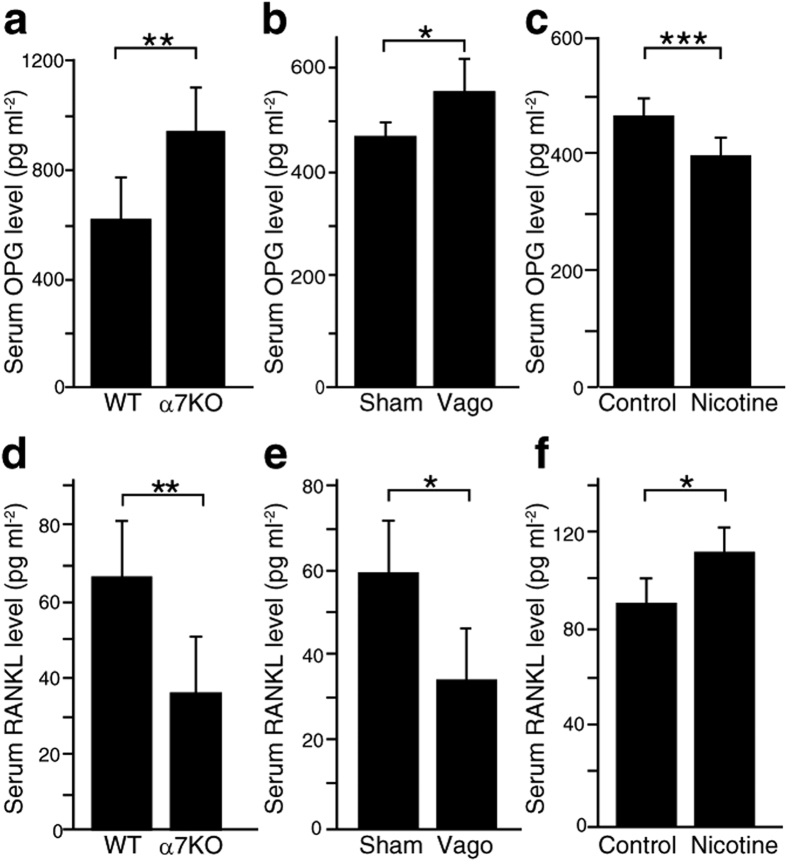
α7nAchR signaling decreases the OPG/RANKL ratio, which is modulated by vagotomy or nicotine administration. (**a–c**) Serum OPG and RANKL were measured by ELISA. (**a**) Levels of serum OPG in wild-type (WT) and α7nAchR KO (α7KO) mice (*n* = 6). (**b**) Levels of serum OPG in sham-operated (Sham) or vagotomized (Vago) WT mice (*n* = 6). (**c**) Levels of serum OPG in WT mice administered nicotine or control saline (*n* = 6). (**d**) Levels of serum RANKL in WT and α7nAchR KO mice (*n* = 6). (**e**) Levels of serum RANKL in sham-operated (Sham) or vagotomized (Vago) WT mice. (*n* = 6). (**f**) Levels of serum RANKL in WT mice administered nicotine or control saline (*n* = 6). All data represent mean serum OPG or RANKL concentrations ± SD (*p < 0.05; **p < 0.01; ***p < 0.001; *n* = 6). Representative data of at least two independent experiments are shown.

To determine how α7nAchR regulates osteoclastogenesis, we analyzed levels of circulating OPG, a RANKL antagonist, given that OPG inhibits osteoclast differentiation and thus positively regulates bone mass^25^,^26^. Serum OPG levels as determined by ELISA significantly increased in *α7nAchR*^−/−^ relative to WT mice (Fig. 3a). To determine whether vagus activity regulates circulating OPG levels, we performed vagotomy in WT mice and observed significantly increased serum OPG levels in vagotomy compared with sham-operated mice (Fig. 3b). Meanwhile, systemic administration of nicotine, a bio-mimetic of acetylcholine, to WT mice downregulated serum OPG levels (Fig. 3c). In contrast to serum OPG, circulating levels of RANKL significantly decreased in *α7nAchR*^−/−^ relative to WT mice (Fig. 3d). Systemic RANKL levels significantly decreased following vagotomy and increased following nicotine administration in WT mice (Fig. 3e and f), suggesting that RANKL and OPG levels are regulated by the vagus nerve via α7nAchR.

**Figure 4:**
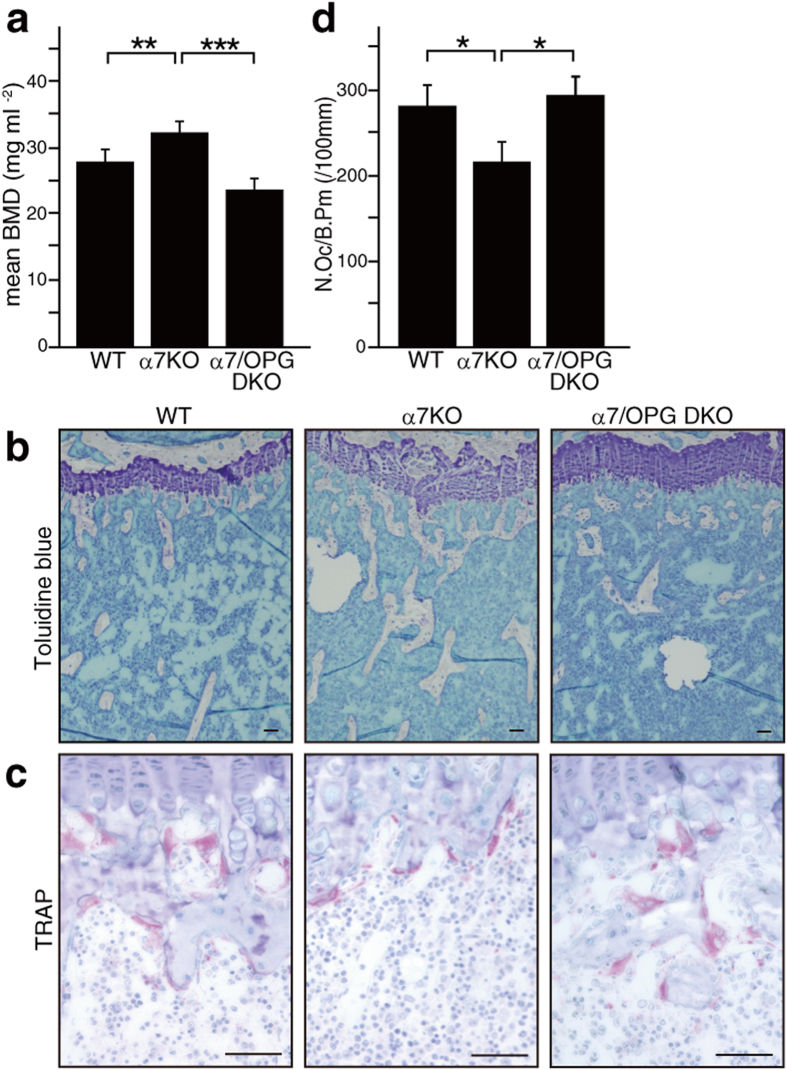
OPG deletion rescues bone phenotypes seen in α7nAchR KO mice *in vivo*. (**a**) Bone mineral density of femurs equally divided longitudinally of 8-week-old wild-type (WT), α7nAchR KO (α7 KO) or α7nAchR/OPG double KO (α7/OPG DKO) mice. Data represent mean bone mineral density ± SD (**p < 0.01; ***p < 0.001; *n* = 5). (**b** and **c**) Histological analysis. Toluidine blue (**b**) and TRAP (**c**) staining of proximal tibiae (Bar = 50 μm). (**d**) Osteoclast number per bone perimeter (N.Oc/B.Pm). Data represent mean N.Oc/B.Pm ± SD (*p < 0.05; *n* = 5). Representative data of two independent experiments are shown.

To determine whether increased bone mass seen in *α7nAchR*^−/−^ mice is due to an elevated serum OPG/RANKL ratio, we crossed *α7nAchR*^−/−^ with OPG-deficient (*Tnfrsf11b*^−/−^) mice to yield α7/OPG DKO mice. In α7/OPG DKO mice, elevated bone mass and inhibited osteoclastogenesis seen in *α7nAchR*^−/−^ mice were significantly rescued (Fig. 4a,b,c and d), suggesting that α7nAchR regulates serum OPG/RANKL ratios and in turn governs osteoclastogenesis.

### Macrophage TNFα regulated by α7nAchR-dependent vagal nerve signaling controls systemic OPG and RANKL levels

**Figure 5:**
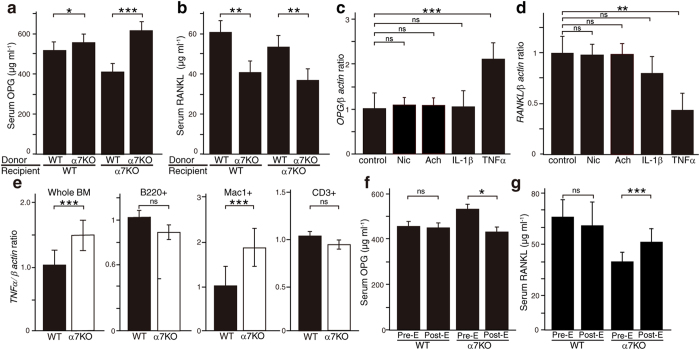
α7nAchR signaling suppressed OPG and elevates RANKL by inhibiting TNFα expression in bone marrow cells. (**a** and **b**) Serum levels of OPG (**a**) and RANKL (**b**) as determined by ELISA in recipient wild-type (WT) or α7AchR KO (α7 KO) mice transplanted with either WT or α7nAchR KO donor cells. Levels were analyzed 12 weeks after transplantation. Data represent mean serum OPG or RANKL levels ± SD (*p < 0.05; **p < 0.01; ***p < 0.001, *n* = 5). (**c** and **d**) *Tnfrsf11b (OPG*) and *Tnfsf11 (RANKL*) expression in MC3T3E1 osteoblastic cells treated with and without nicotine (1 μM, Nic), acetylcholine (1 μM, Ach), IL-1β (10 ng/ml) or TNFα (10 ng/ml) for 24 hours, as determined by realtime PCR. Data represent mean *OPG* (**c**) or *RANKL* (**d**) expression relative to *β-actin* ± SD (**p < 0.01; ***p < 0.001, NS: not significant, *n* = 3). (**e**) *Tnfα* expression as determined by realtime PCR in cells from whole bone marrow, B220 + B cells, Mac1 + monocyte/macrophages or CD3 + T cells fractionated by flow cytometry in wild-type (WT) and α7nAchR KO mice. Data represent mean *Tnfα* expression relative to *β-actin* ± SD (*n* = 3). (**f** and **g**) Serum levels of OPG (**f**) and RANKL (**g**) as determined by ELISA in WT or α7nAchR KO mice before (Pre) or three weeks after (Post) administration of Etanercept (E), a TNFα inhibitor. Data represent mean OPG or RANKL levels ± SD (*p < 0.05; ***p < 0.001; NS: not significant, *n* = 6). Representative data of at least two independent experiments are shown.

Next, we asked which cells were responsible for regulating circulating OPG and RANKL levels in response to vagus nerve signaling (Fig. 5). α7nAchR is abundantly expressed in brain and heart^30^,^31^, and thus we initially asked whether these tissues might regulate OPG and RANKL levels. To do so, we undertook analysis of bone-related phenotypes using bone marrow (BM) transfer of WT or *α7nAchR*^−/−^ BM cells into α7*nAchR*^−/−^ or WT mice. We observed that serum OPG levels significantly decreased following transfer of WT BM into mutant mice relative to transplantation with α7*nAchR*^−/−^ BM cells (Fig. 5a). In contrast, *α7nAchR*^−/−^ BM cell transfer into WT mice significantly elevated serum OPG levels compared to WT mice transplanted with WT BM cells (Fig. 5a). These observations suggest that BM cells are responsible for OPG production via α7nAchR. To determine which cells regulated OPG and RANKL expression in BM cells, we fractionated WT and *α7nAchR*^−/−^ BM cells into CD3-positive T cells, B220-positive B cells and Mac1-positive monocyte/macrophage cells, and analyzed *Tnfrsf11b (OPG)* expression by realtime PCR in each; however, *Tnfrsf11b* expression was not detected in any of the three populations (data not shown). Serum RANKL levels were downregulated by transfer of *α7nAchR*^−/−^ BM cell into either WT or *α7nAchR*^−/−^ mice (Fig. 5b), strongly suggesting that BM cells regulate both OPG and RANKL levels via α7nAchR indirectly.

IL-1β and TNFα are both reportedly upregulated in α7*nAchR*^−/−^ mice^4^, and we found that *Tnfrsf11b (OPG)* expression was significantly elevated by TNFα but not IL-1β in MC3T3-E1 osteoblastic cells *in vitro* (Fig. 5c). Similarly, we also found that *Tnfsf11 (RANKL)* expression was significantly downregulated by TNFα in MC3T3-E1 cells (Fig. 5d). *Tnfα* mRNA expression was specifically and significantly higher in whole BM cells and in Mac1-positive cells sorted from α7nAchR-deficient compared to WT mice (Fig. 5e). We then administered the TNFα-inhibitor, Etanercept, a soluble receptor of TNFα, to mice of either genotype and observed downregulation of serum OPG levels in *α7nAchR*^−/−^ but not in WT mice (Fig. 5f). By contrast, RANKL levels were significantly elevated following Etanercept administration to *α7nAchR*^−/−^ but not WT mice (Fig. 5g).

### Elevated bone mass in α7nAchR-deficient mice is reversed by TNFα deletion

**Figure 6:**
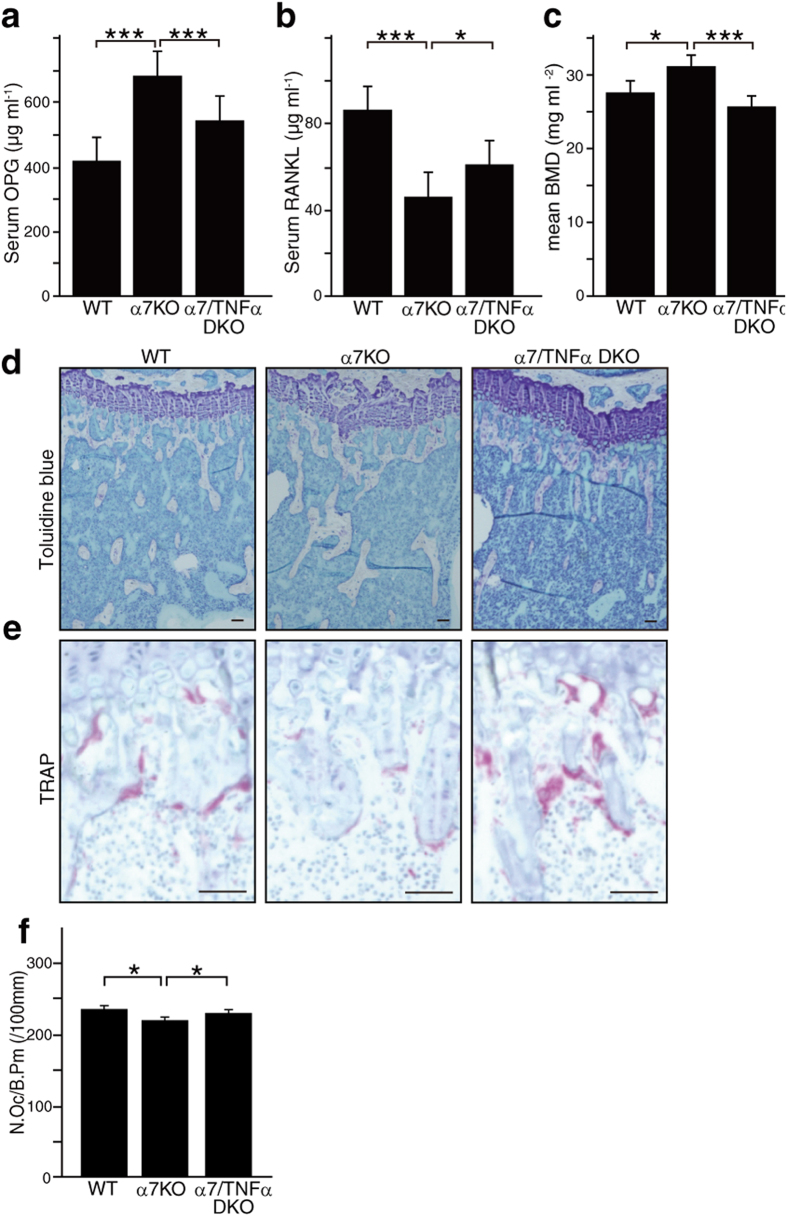
Elevated TNFα seen in α7nAchR KO mice is required to increase bone mass and the OPG/RANKL ratio. (**a** and **b**) Serum levels of OPG (**a**) and RANKL (**b**) as determined by ELISA in wild-type (WT), α7nAchR KO (α7 KO) and α7nAchR/TNFα doubly deficient (α7/TNFα DKO) mice. Data represent mean serum OPG or RANKL levels ± SD (*p < 0.05; ***p < 0.001, *n* = 5). (**c**) Bone mineral density (BMD) of femurs divided equally longitudinally from 8-week-old wild-type (WT), α7nAchR KO (KO) or α7/TNFα DKO mice. Data represent means ± SD of BMD (*p < 0.05; ***p < 0.001, *n* = 5). (**d** and **e**) Histological analysis. Toluidine blue (**d**) and TRAP staining (**e**) of the proximal tibiae (Bar = 50 μm). (**f**) Osteoclast number per bone perimeter (N.Oc/B.Pm). Data represent mean N.Oc/B.Pm ± SD (*p < 0.05, *n* = 5). Representative data of two independent experiments are shown.

Finally, we crossed α7*nAchR*^−/−^ mice with *Tnfα*^−/−^ mice to yield α7nAchR and TNFα-doubly deficient (α7/TNFα DKO, *α7nAchR*^−/−^*Tnfα*^−/−^) mice. Resulting double-knockout mice showed significantly reduced serum OPG and elevated RANKL, a reversal of what is seen in *α7nAchR*^−/−^ single knockout mice (Fig. 6a and b). *α7nAchR*^−/−^*Tnfα*^−/−^ mice also showed normal bone mass and osteoclast formation, unlike *α7nAchR*^−/−^ mice (Fig. 6c–f).

Taken together, our results suggest that vagus nerve stimulation enhances osteoclast formation and reduces bone mass by suppressing serum OPG levels and elevating serum RANKL levels through inhibition of α7nAchR-dependent macrophage TNFα expression.

## Discussion

**Figure 7:**
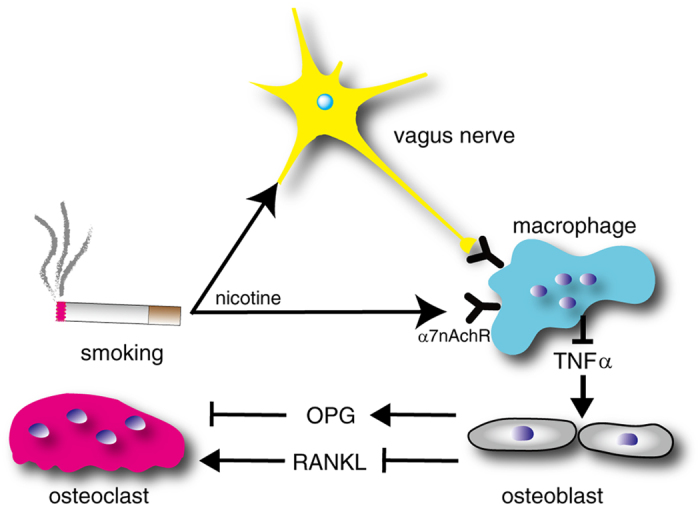
Schematic model showing the role of vagus nerve activity and nicotine in regulating the OPG/RANKL ratio and bone homeostasis. Proposed regulation of osteoclastogenesis via the vagus nerve/macrophage axis. Vagal nerve activity potentially stimulated by either physiologically or nicotine activation due to smoking via α7nAchR inhibits TNFα expression in macrophages, which in turn, induces OPG and suppresses RANKL expression in osteoblasts.

Bone homeostasis is orchestrated by a combination of local and systemic factors physiologically and perturbed in pathological inflammatory conditions. Here, we demonstrate that bone homeostasis is regulated by vagus nerve activity via α7nAchR. Our model suggests that osteoclast formation is enhanced by down-regulation of OPG levels and up-regulation of serum RANKL levels following inhibition of macrophage TNFα expression, the latter controlled by α7nAchR-dependent vagus nerve activity (Fig. 7). α7nAchR loss elevated TNFα expression in macrophages, leading to elevated OPG and reduced RANKL levels in sera, and turn inhibiting osteoclast formation and increasing bone mass. Inhibition of TNFα in *α7nAchR*^−/−^ mice reversed the perturbed OPG/RANKL ratio status and normalized bone mass. Vagotomy elevated OPG and decreased RANKL levels in WT mouse sera, a phenotype similar to that seen in *α7nAchR*^−/−^ mice. Finally, nicotine administration reduced OPG and increased RANKL levels in serum of WT mice.

The vagus nerve controls heart, brain and gut function and/or development, but no obvious abnormality is seen in these organs in α7nAchR-deficient mice^1^. Instead, α7nAchR reportedly functions to inhibit inflammation and septic reactions^4^,^8^,^9^, and thus vagus nerve control of an anti-inflammatory axis likely suppresses excessive immune responses. Here, we show that vagal nerve activity is required to regulate bone homeostasis by controlling Mac1-positive cell expressing TNFα-mediated OPG and RANKL expression in physiological conditions. Elevated serum OPG levels seen in α7nAchR-deficient mice were partially but significantly restored by deletion of the gene encoding TNFα, suggesting that α7nAchR regulates OPG levels through factors other than TNFα and IL-1β. Since our bone marrow transplantation analysis demonstrated that bone marrow-derived Mac1-positive cells regulate serum OPG levels, cholinergic anti-inflammatory signaling in those cells must control those levels. This cholinergic anti-inflammatory pathway reportedly functions in macrophages^4^. Mac1 is reportedly expressed in monocytes/macrophages^32^, and these cells are considered regulate serum OPG levels via α7nAchR. Mac1 is also expressed in neutrophils and natural killer cells^33^,^34^. Further studies are needed to determine the lineage of Mac1-positive cells that regulates TNFα via α7nAchR.

Moreover, TNFα promotes RANKL expression in osteoblastic cells^35^,^36^; however, in this study, we showed that RANKL expression in osteoblastic cells is inhibited by TNFα. TNFα activates MAPK and NFκB pathways^35^,^36^, and our data suggest that the MAPK rather than the NFκB pathway contribute controls OPG by TNFα signaling (Fig. S1).

α7nAchR is expressed in osteoblasts^37^, an observation we confirmed here (Fig. S2a and S2b). Whether α7nAchR-deficient mice exhibit bone phenotypes is controversial: some authors reported normal bone mass based on 3D micro CT analysis^10^, while others have shown elevated bone mass based on micro CT analysis^11^. Here, we show relatively elevated bone mass in α7nAchR-deficient based on DEXA, micro CT and bone histomorphometric analysis. Furthermore, in the absence of nicotine or acetylcholine stimulation, others previously showed that osteoclastogenesis is inhibited in α7nAchR-deficient compared to wild-type cells *in vitro*^11^. However, our data indicates that bone homeostasis is regulated via α7nAchR systemically rather than locally through serum OPG and RANKL. Nicotine is known to stimulate calcium signals in chondrocytes^37^. We have preliminary data showing that nicotine or acetylcholine stimulates calcium signals in macrophages; however, nicotine stimulation reportedly inhibits RANKL-induced calcium oscillation^11^. Thus, mechanisms used by α7nAchR to regulate a cholinergic anti-inflammatory pathway in macrophages have not been clarified, and further studies are required to determine how α7nAchRs inhibit inflammation.

The sympathetic and parasympathetic nervous systems cooperatively regulate functions of various organs. However, sympathetic activity reportedly inhibits osteoblastogenesis, leading to bone loss^18^. Here, we showed that parasympathetic activity stimulates osteoclast formation and reduces bone mass. Thus both the sympathetic and parasympathetic systems apparently reduce bone mass via inhibiting bone-formation and stimulating bone-resorption, respectively.

Inflammation is a risk factor for bone loss, and patients with inflammatory disease such as RA exhibited osteoporosis^37^. TNFα is a major inflammatory cytokine upregulated in RA patients and is implicated in RA pathogenesis^38^. Transgenic mice overexpressing human TNFα exhibit RA-like phenotypes, such as joint inflammation and bone erosion due to increased osteoclast formation^17^. Indeed, treatment with biologics targeting TNFα is effective in treating RA patients^15^,^39^. TNFα expression is also promoted by pathologic conditions such as bacterial or viral infection or injury, while negative feedback regulation of TNFα-induced inflammation is mediated by induction of anti-inflammatory cytokines^40^. We show here that vagus nerve activity inhibits TNFα expression by macrophages via α7nAchR, and the pathology in both collagen-induced arthritis or sepsis models is more severe in *α7nAchR7*^−/−^ than in control mice^8^,^9^. Our study suggests that in the bone system, TNFα-mediated OPG induction and RANKL suppression likely represent a feedback mechanism to protect from excessive bone loss.

TNFα reportedly promotes osteoclast formation directly without RANKL^32^,^41^. However, bone phenotypes have not been reported in TNFα-deficient mice, suggesting that TNFα does not play a central role in regulating bone mass in physiological conditions but rather enhances bone erosion in pathological contexts. We show here that bone mass is elevated even under high TNFα conditions in *α7nAchR*^−/−^ mice, indicating that TNFα-induced OPG expression and RANKL suppression is dominant over TNFα-promoted bone loss in physiological conditions.

OPG is a decoy soluble receptor of RANKL and blocks osteoclast differentiation signals to the RANK receptor. In physiological conditions, OPG and RANKL expression is controlled in osteoblastic cells by osteotropic factors such as 1,25(OH)_2_D_3_ and PGE2^24^. In contrast, OPG expression reportedly increases in pathological conditions such as LPS administration^42^. RANKL expression is also stimulated by inflammatory cytokines^29^. Recently, OPG expression was reported to be induced via the PHD-HIF2α axis^43^. Here, we show that the vagus nerve-TNFα axis regulates serum OPG and RANKL levels.

The natural ligand of nAchRs is acetylcholine, an activity mimicked by nicotine. Smoking is a known risk factor for development of osteoporosis/osteopenia^12^, and is implicated as a negative factor for fracture healing^44^. In the current study, we show for the first time that administration of nicotine to WT mice significantly reduced levels of circulating OPG and elevated levels of RANKL. This observation may explain, at least in part, the negative effect of nicotine on bones. Smoking is also seen as a risk factor for RA development^45^.

Taken together, our data present new insight into regulation of bone homeostasis by the vagus nerve and how this interaction is mediated by inflammatory cytokine signaling.

## Methods

### Mice

*α7nAchR*^*+/−*^, *α7nAchR*^−/−^, *Tnfrsf11b*^−/−^ and *Tnf*α^−/−^ mice were maintained as described^35^,^37^,^46^. *α7nAchR*^−/−^ and *Tnfrsf11b*^−/−^ mice or *α7nAchR*^−/−^ and *Tnf*α^−/−^ mice were crossed to generate *α7nAchR*^−/−^*Tnfrsf11b*^−/−^ or *α7nAchR*^−/−^*Tnf*α^−/−^ mice, respectively.

Bone marrow transplantation was performed as previously described^47^. Briefly, 1 × 10^6^ of bone marrow cells from 8-week-old donor (WT or *α7nAchR*^−/−^) mice were transplanted intravenously into 10-week-old recipients (WT or *α7nAchR*^−/−^), which were irradiated at a lethal dose immediately before transplant.

For bone mineral density (BMD) analysis, femurs were removed from WT, *α7nAchR*^−/−^, *Tnfrsf11b*^−/−^, *α7nAchR*^−/−^*Tnfrsf11b*^−/−^ or *α7nAchR*^−/−^*Tnf*α^−/−^ mice, fixed with 70% ethanol and subjected to dual energy x-ray absorptiometric (DEXA) scanning to measure BMD (mg/cm^2^) at 20 proximal to distal points using a DCS-600R system (Aloka Co. Ltd, Tokyo, Japan). To evaluate bone architecture, micro-computed tomography (μCT) analysis was performed. Bone morphometric analysis and tartrate-resistant acid phosphatase (TRAP) staining were performed in tibiae from WT or *α7nAchR*^−/−^ mice as described^48^.

Animals were maintained under specific pathogen-free conditions in animal facilities certified by the Keio University animal care committee. Animal protocols were approved by that committee and carried our in accordance with the committee’s guidelines.

### Vagotomy

Bilateral subdiaphragmatic vagotomy or sham surgery was performed in 8-week-old WT mice as described^49^. Briefly, the stomach and lower esophagus were exposed by laparotomy. The stomach was gently retracted downward beneath the diaphragm to visualize both vagal trunks and then the bilateral vagus nerve was removed. For pyloroplasty to prevent gastric stasis, an incision was made parallel to the axis of the pylorus through the pyloric sphincter, and the pylorus wall was reconstructed by sutures perpendicular to the pylorus axis. The stomach was returned to its normal position, and incisions were closed. For sham-operated animals, after opening the abdominal cavity, pyloroplasty only was performed.

### Serum OPG and RANKL assay

Sera were collected from 8-week-old WT, *α7nAchR*^−/−^, *Tnfrsf11b*^−/−^, *α7nAchR*^−/−^*Tnfrsf11b*^−/−^ or α7*nAchR*^−/−^*Tnf*α^−/−^ mice, and recipients transplanted with bone marrow cells twelve-week-after transplantation. Sera were also collected from vagotomized or nicotine administrated wild-type mice, or from mice administered 25 mg/kg of Etanercept (Embrel^®^. Pfizer. Tokyo. Japan) twice a week for three weeks. OPG or RANKL levels in sera were examined by ELISA according to the manufacture’s protocol (R&D).

### Flow cytometry

Bone marrow mono-nuclear cells isolated from WT or *α7nAchR*^−/−^ mice were stained with FITC-conjugated anti-CD3, B220 or Mac1. Then, CD3-positive T cells, B220-positive B-cells and Mac1-positive monocyte/macrophage fractions were sorted separately using the B & D FACS Aria^TM^ 2 system.

### Immunofluorescence

Paraffin sections of tibia were deparaffinized and subjected to antigen retrieval as described^50^. Sections were then stained with rabbit anti-α7nAchR (1:00 Santa Cruz Biotechnology) followed by Alexa488-conjugated goat anti-mouse Ig’ (1:200; Invitrogen, Carlsbad, CA). DAPI (1:750; Wako Pure Chemicals Industries, Osaka, Japan) served as a nuclear stain.

### Reagents

Macrophage colony-stimulating factor (M-CSF) and recombinant soluble receptor activator of nuclear factor κ–B ligand (RANKL) were purchased from Kyowa Hakko Kirin Co. (Tokyo, Japan) and Pepro-Tech Ltd. (Rocky Hill, NJ), respectively. Nicotine and acetylcholine were obtained from Sigma-Aldrich (St. Louis, MO).

### Cell culture

To assess osteoclast formation *in vitro*, bone marrow cells were isolated from femurs and tibiae of WT and *α7nAchR*^−/−^ mice and cultured 3 days in α-modified Eagle’s minimum essential medium (Sigma-Aldrich Co.) containing 10% heat-inactivated FBS (JRH Biosciences, Lenexa, KS, USA) and GlutaMax (Invitrogen Corp.) with M-CSF (50 ng/ml). M-CSF-dependent adherent cells were then collected as osteoclast progenitors, and 5 × 10^4^ cells were plated per well of 96-well culture plates and cultured with M-CSF (50 ng/ml) and RANKL (25 ng/ml) in the presence or absence of nicotine (1 μM) or acetylcholine (1 μM) for five days. Osteoclastogenesis was assessed by evaluation of TRAP and May-Giemsa staining under a microscope (BZ-9000, Keyence Co., Tokyo, Japan), and TRAP-positive cells containing more than three nuclei were scored as osteoclasts^51^.

Primary osteoblasts were isolated from newborn mouse calvariae as described^52^. Osteoblastic MC3T3E1 cells were cultured in α-MEM containing 10% FCS in the presence or absence of acetylcholine (1 μM), nicotine (1 μM), IL-1β (10 ng/ml) or TNFα (10 ng/ml) for 24 h.

### Quantitative PCR

Total RNAs were isolated from cultured or sorted cells using the RNeasy mini kit (Qiagen), and cDNA was synthesized with oligo (dT) primers and reverse transcriptase (Takara Bio Inc). Quantitative PCR was conducted using the SYBR Premix ExTaq II (Takara Bio Inc., Otsu, Shiga, Japan) with a DICE Thermal cycler (Takara Bio Inc.) according to the manufacturer’s instructions. *β-actin* expression was analyzed as an internal control. Primers for *β-actin, Ctsk, Nfatc1, Tnfα, Tnfrsf11b (OPG) and Tnfsf11 (RANKL*) are as follows.

*β-actin*-forward: 5′-TGAGAGGGAAATCGTGCGTGAC-3′

*β-actin*-reverse: 5′-AAGAAGGAAGGCTGGAAAAGAG-3′

*Ctsk*-forward: 5′-ACGGAGGCATTGACTCTGAAGATG-3′

*Ctsk*-reverse: 5′-GGAAGCACCAACGAGAGGAGAAAT-3′

*Nfatc1*-forward: 5′-CAAGTCTCACCACAGGGCTCACTA-3′

*Nfatc1*-reverse: 5′-GCGTGAGAGGTTCATTCTCCAAGT-3′

*Tnfα*-forward: 5′-CTTCTGTCTACTGAACTTCGGG-3′

*Tnfα*-reverse: 5′-CAGGCTTGTCACTCGAATTTTG-3′

*Tnfrsf11b (OPG)*-forward: 5′-GACCACAATGAACAAGTGGCTGT-3′

*Tnfrsf11b (OPG)*-reverse: 5′-CCAGTTTCTGGGTCATAATGCAA-3′

*Tnfsf11 (RANKL)*-forward: 5′-GCATCGCTCTGTTCCTGTACTTT-3′

*Tnfsf11 (RANKL)*-reverse: 5′-CGTTTTCATGGAGTCTCAGGATT-3′

### Statistical analyses

Statistical analysis was performed using the unpaired two-tailed Student’s *t*-test (*p < 0.05; **p < 0.01; ***p < 0.001; NS, not significant, throughout the paper). All data are expressed as means ± SD.

## Additional Information

**How to cite this article**: Mito, K. *et al*. The nicotinic acetylcholine receptor α7 subunit is an essential negative regulator of bone mass. *Sci. Rep.* **7**, 45597; doi: 10.1038/srep45597 (2017).

**Publisher's note:** Springer Nature remains neutral with regard to jurisdictional claims in published maps and institutional affiliations.

## Supplementary Material

Supplementary Figures
