# Supplementary material for: The nicotinic acetylcholine receptor α7 subunit is an essential negative regulator of bone mass
Source: Sci Rep. 2017 Mar 28;7:45597. doi: 10.1038/srep45597 (PMC5368561; doi:10.1038/srep45597)
Supplement: Supplementary Figures [file srep45597-s1.pdf]

The nicotinic acetylcholine receptor α7 subunit is an essential negative regulator of bone mass

Kazuaki Mito[1], Yuiko Sato[1,2], Tami Kobayashi[1,2], Kana Miyamoto[1], Eriko Nitta[4], Atsushi Iwama[4], Morio Matsumoto[1], Masaya Nakamura[1], Kazuki Sato[1] and Takeshi Miyamoto[1,3,*]

[1]Department of Orthopedic Surgery, [2]Department of Musculoskeletal Reconstruction and Regeneration Surgery, [3]Department of Advanced Therapy for Musculoskeletal Disorders, Keio University School of Medicine, 35 Shinano-machi, Shinjuku-ku, Tokyo 160-8582, Japan

[4]Department of Cellular and Molecular Medicine, Graduate School of Medicine, Chiba University, 1-8-1 Inohara, Chuo-ku, Chiba 260-8670, Japan

Supplementary Figure Legends

Figure S1. MAPKs regulate OPG expression by TNFα in osteoblastic cells

MC3T3E1 cells were cultured with TNFα (10 ng/ml) in the presence or absence of SB203580 (10μM, p38 inhibitor), SP600125 (10μM, JNK inhibitor), U0126 (10μM, MEK and thus ERK inhibitor) or DHMEQ (1 μg/ml, NFκB inhibitor) for 24h. *Tnfrsf11b* (*OPG*) and *Tnfsf11* (*RANKL*) expression was then determined by realtime PCR. Data represent mean *OPG* (**a**) or *RANKL* (**b**) expression relative to *β-actin* ± SD (**$p<0.01$, NS: not significant, n=3).

Figure S2. Osteoblastic cells express α7nAchR.

(a) Paraffin sections of tibia were stained with rabbit anti-α7nAchR followed by Alexa488-conjugated goat anti-mouse Ig'. DAPI served as nuclear stain. Sections were observed by fluorescence microscopy. Osteoblastic lining cells but not intra-cortical osteocytes stain positively for α7nAchR. CB, cortical bone; BM, bone marrow cavity. Arrows indicate α7nAchR-positive osteoblastic lining cells. Bar=20 μm. (b) *α7nAchR* expression as analyzed in bone marrow-derived macrophages (BMM), osteoclasts (OCL) and primary osteoblasts by realtime PCR. Data represent mean *α7nAchR* expression relative to *β-actin* ± SD (***$p<0.001$; NS: not significant, n=3).

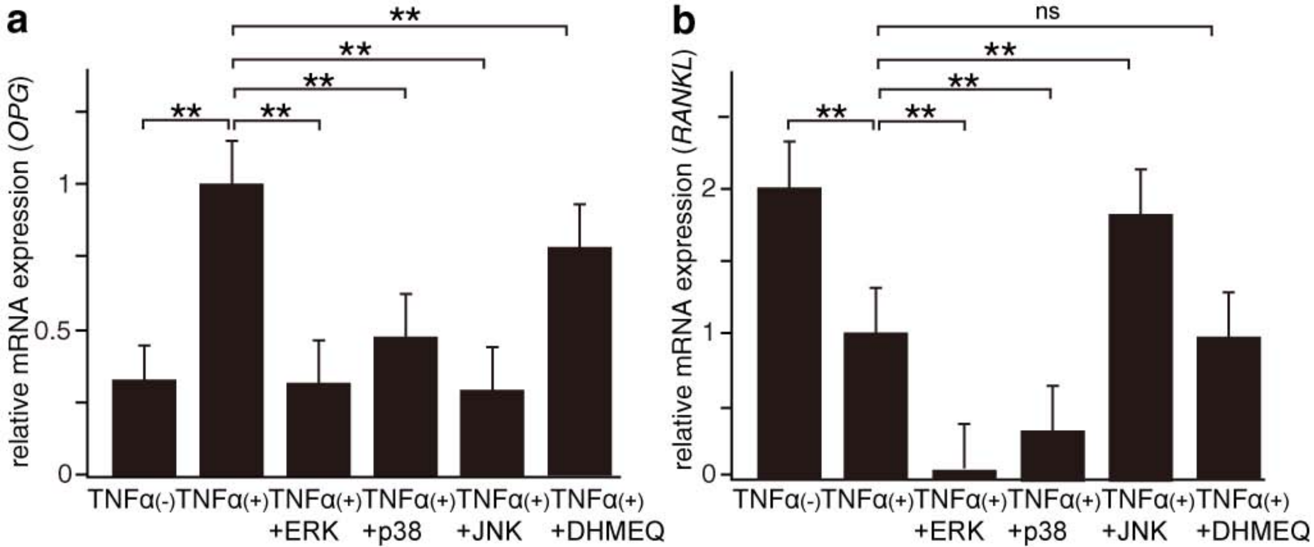

Figure S1. Mito et al.

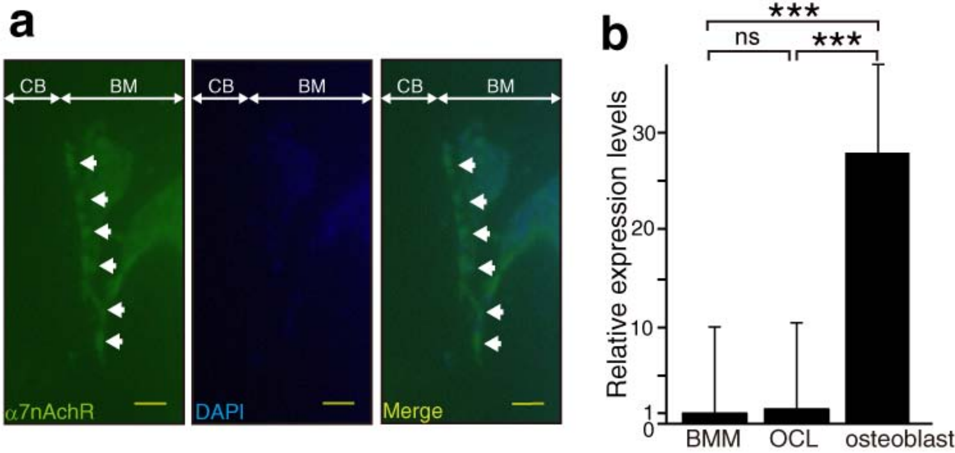

Figure S2. Mito et al.
